# Associations of facility-level antibiotic use and hospital-onset *Clostridioides difficile* infection in US acute-care hospitals, 2012–2018

**DOI:** 10.1017/ice.2021.151

**Published:** 2022-08

**Authors:** Sophia V. Kazakova, James Baggs, Sarah H. Yi, Sujan C. Reddy, Kelly M. Hatfield, Alice Y. Guh, John A. Jernigan, L. Clifford McDonald

**Affiliations:** Division of Healthcare Quality Promotion, National Center for Emerging and Zoonotic Infectious Diseases, Centers for Disease Control and Prevention (CDC), Atlanta, Georgia

## Abstract

Previously reported associations between hospital-level antibiotic use and hospital-onset *Clostridioides difficile* infection (HO-CDI) were reexamined using 2012–2018 data from a new cohort of US acute-care hospitals. This analysis revealed significant positive associations between total, third-generation, and fourth-generation cephalosporin, fluoroquinolone, carbapenem, and piperacillin-tazobactam use and HO-CDI rates, confirming previous findings.

Although the number of cases of *Clostridioides difficile* infection (CDI) in the United States has decreased in recent years,^1,2^ disease burden remains high,^3^ and the Centers for Disease Control and Prevention has recently declared CDI an urgent public health threat.^4^ Decreases in hospital-onset CDI (HO-CDI) rates have occurred despite relatively stable national rates of overall inpatient antibiotic use.^5,6^ Previously, we reported the association between hospital-level antibiotic use rates and HO-CDI rates in acute-care hospitals (ACHs) from 2006 to 2012.^7^ In this analysis, we reexamined the cross-sectional and temporal associations between hospital-level antibiotic use and HO-CDI rates using more recent data from a separate cohort of ACHs.

## Methods

We used adult hospital discharge and inpatient charge records for antibiotic use, CDI tests and treatment from ACHs contributing to the Premier Healthcare Database from January 1, 2012, to December 31, 2018. A case of HO-CDI was defined as a hospital discharge with an *International Classification of Diseases* (ICD) code specifying enterocolitis due to *Clostridioides difficile* (ICD-9 008.45 or ICD-10 A04.7, A04.71, or A04.72) in any secondary diagnostic position and inpatient treatment with metronidazole (parenteral or oral), fidaxomicin, or vancomycin (oral) initiated on hospital day 3 or later after admission. Facility-level monthly rates of HO-CDI were calculated per 10,000 patient days (PD). Hospital antibiotic use was measured by days of therapy (DOT) per 1,000 PD and examined through monthly rates of total antibiotic use and use of 7 antibiotic classes: fluoroquinolones, third- and fourth-generation cephalosporins (cephalosporins), piperacillin-tazobactam, carbapenems, β-lactam/β-lactamase inhibitor combination (excluding piperacillin-tazobactam), clindamycin, and penicillins. In addition, we evaluated combined use of 3 high-risk antibiotic classes: fluoroquinolones, cephalosporins, and carbapenems.

The cross-sectional relationships between facility-level rates of HO-CDI and antimicrobial use were examined through multivariable generalized estimating equation (GEE) models accounting for autocorrelation between monthly HO-CDI rates within a facility, negative binomial distribution, annual trend, month of discharge, and included a natural log of patient day as offset. All GEE models adjusted for hospital confounders: urban or rural location, bed size, teaching status, census division, primary nucleic acid amplification test (NAAT) utilization, proportion of patients aged ≥65 years, average Gagne comorbidity score,^8^ patient case-mix index, proportion of discharges with a surgical diagnosis-related group (DRG), and community-onset CDI (CO-CDI) cases per 100 discharges. A case of CO-CDI was defined as a discharge with a ICD-9 or CD-10 diagnosis code for CDI in the primary position, no prior hospitalization in the same facility in 28 days, and inpatient CDI treatment initiated at any time during hospitalization. NAAT testing was assessed using inpatient charges for *C. difficile* tests where each test was categorized as NAAT if its description contained “NAAT.” “PCR,” “amplified,” or “DNA.” All other *C. difficile* tests were categorized as “non-NAAT.” NAAT primary utilization was defined quarterly as a binary variable. If all *C. difficile* tests in a given quarter included an NAAT test, a hospital was considered to have primary NAAT utilization; otherwise, a hospital was considered not to have primary NAAT utilization.

Temporal associations between HO-CDI rate and antibiotic use were assessed for subsets of ACHs that achieved the following targeted decreases in antibiotic use over 2 consecutive years: <10% decrease, ≥30% decrease, ≥20% decrease, and ≥10% decrease (groups are cumulative and inclusive). Temporal trends in HO-CDI rates were assessed using GEE models similar to the cross-sectional analysis with the addition of adjustment for seasonality.

## Results

During 2012–2018, among 921 participating hospitals, the median hospital-level HO-CDI rate was 6.7 per 10,000 PD (interquartile range [IQR], 4.2–9.5); the median CO-CDI rate was 0.26 per 100 discharges (IQR, 0.16–0.38) (Supplementary Table 1 online). More than half of hospital quarters (52.5%) had primary NAAT utilizations (Supplementary Fig. 1 online), and primary NAAT utilization peaked at 57% in 2015 (Supplementary Fig. 2 online). In a cross-sectional multivariable analysis, overall antibiotic use was significantly associated with the facility-level HO-CDI rate. For every 50 DOT per 1,000 PD increase in antibiotic use, there was a 2.8% increase in the HO-CDI rate (rate ratio [RR], 1.028; *P* < .001). In a class-specific model, 10 DOT per 1,000 PD increases in the use of carbapenems, cephalosporins, and piperacillin-tazobactam were each independently associated with 1.3%, 0.6%, and 1.1% increases in the HO-CDI rate, respectively (Supplementary Table 2 online). In all models, primary NAAT utilization was associated with a 16% higher HO-CDI rate compared with hospitals without primary NAAT utilization (Supplementary Table 2).

**Fig. 1.**
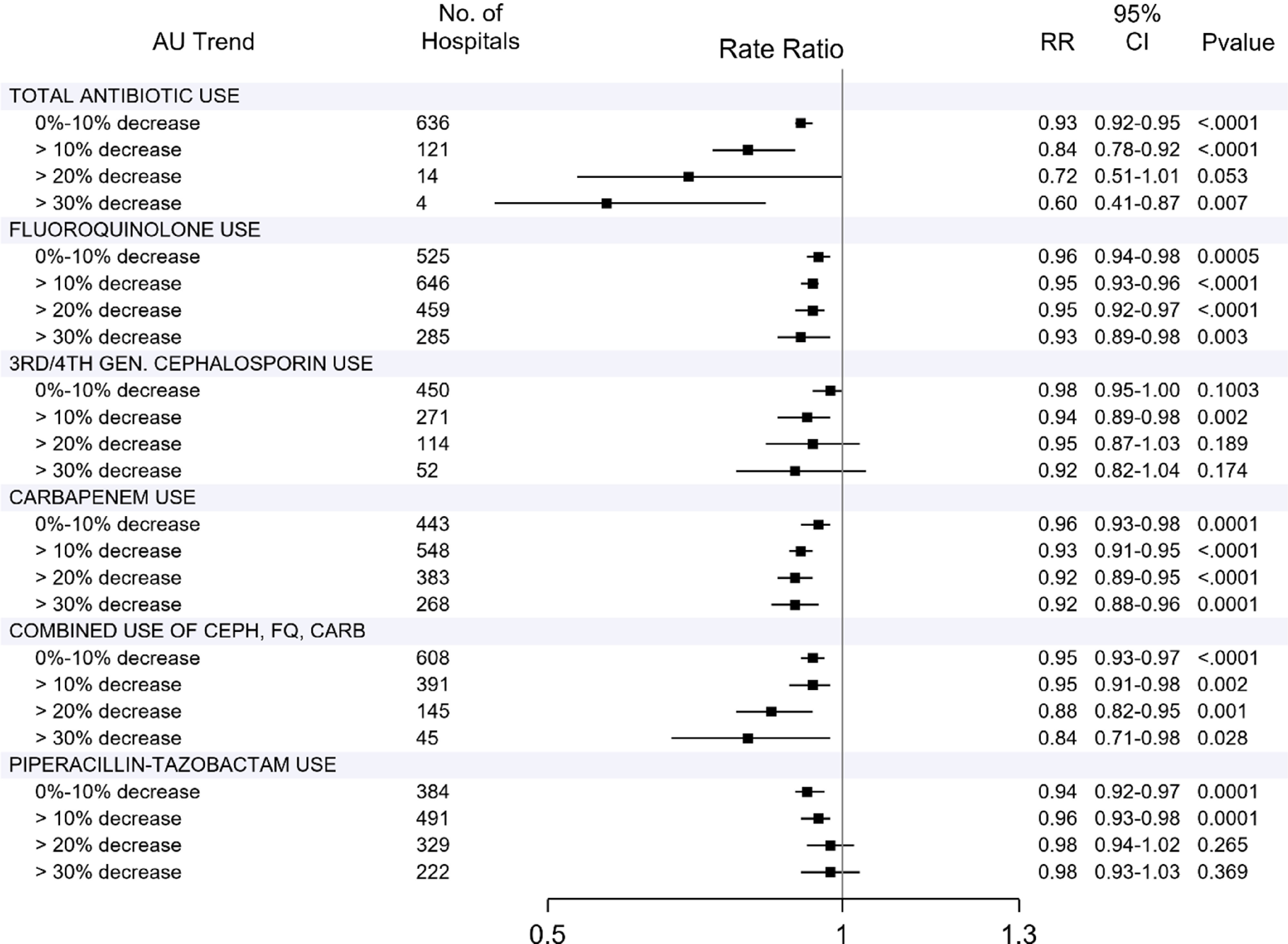
Analysis of trends in hospital-onset *C. difficile* infection (HO-CDI) rates in the US acute-care hospitals by level of decreased antibiotic use, 2012–2018 Premier Healthcare Database. Adjusted rate ratios and 95% confidence intervals for the temporal trends in HO-CDI in the 24 not-mutually exclusive groups of ACHs achieving targeted decreases in antibiotic use over 2 consecutive years; >10%, >20%, >30% means ≥10%, ≥20%, ≥30% decreases. Temporal trends in HO-CDI were assessed using generalized estimating equation (GEE) models that assumed negative binomial distribution of HO-CDI, autoregressive correlation of repeated measurements within ACHs, offset by patient-days and adjusted for seasonality, patient (case-mix category, community-onset CDI rate, proportion of patients aged 65 or older, average Gagne comorbidity score, and proportion of surgical patients) and hospital (primary NAAT utilization, urban vs. rural, bed size, teaching status, census division) characteristics. Note. HO-CDI, hospital-onset *Clostridioides difficile* infection; AU, antibiotic use; RR, rate ratio; FQ, fluoroquinolone; CEPH, third- and fourth-generation cephalosporins; CARB, carbapenems.

Adjusted trends in HO-CDI rate ratios for each group of antibiotic use decreases are represented in Figure 1. Notably, the 4 hospitals that decreased total antibiotic use ≥30% demonstrated a 40% decrease in their HO-CDI rates (RR, 0.60; 95% confidence interval [CI], 0.41–0.87). Decreases in fluoroquinolone and carbapenem use corresponded with annual decreases in HO-CDI rates of 4%–7% and 4%–8%, respectively. Decreases in the combined use of cephalosporins, fluoroquinolones, and carbapenems corresponded with annual decreases in the HO-CDI rate of 4%–16%.

## Discussion

In this ecologic analysis, we used 2012–2018 data from a large cohort of ACHs to identify significant associations between facility-level HO-CDI rates and antibiotic utilization. Specifically, higher levels of total antimicrobial use as well as use of third- and fourth-generation cephalosporins, carbapenems, and piperacillin-tazobactam were associated with higher rates of HO-CDI. Decreases in total antibiotic use, fluoroquinolones, carbapenems, and combined use of fluoroquinolones, third- and fourth-generation cephalosporins, and carbapenems corresponded with decreases in HO-CDI. This analysis builds upon our previous investigation by confirming associations identified in a different hospital cohort over a different period and by adjusting for a confounder not previously available, that is, hospital-level use of a more sensitive NAAT diagnostic test. Although NAAT utilization was derived from administrative data, which is subject to several previously described limitations,^7^ its temporal association with HO-CDI indicates the importance of including this factor in HO-CDI models (Supplementary Fig. 2 online).

Like our previous study, the largest reductions in HO-CDI rates were identified in a small set of ACHs that decreased their total antibiotic use by 30%. These findings are also similar to those of a study by Rhea et al^9^ showing that a simulated 30% reduction in antibiotics prescribed across all inpatient and outpatient locations of a regional network was associated with a 17% decrease in HO-CDI. Given reports of widespread inappropriate use, reductions in total antibiotic use of this magnitude are potentially feasible, particularly among hospitals with high antibiotic use.^10^ Although decreasing total antibiotic use is an attractive goal, our findings suggest that important reductions in HO-CDI rates may be achieved with a more targeted approach that focuses on combined use of cephalosporins, fluoroquinolones, and carbapenems. Hospitals with a ≥30% decrease in the combined use of those antibiotics observed a 16% decrease in the HO-CDI. This approach may also help prevent substitution of fluoroquinolones by other broad-spectrum antibiotics such as cephalosporins, an occurrence evident in recent reports on antibiotic use trends.^5,6^ Although specific reasons for and factors driving the changes in antibiotic use in our hospital cohort were beyond the scope of this investigation, we found that decreased antibiotic use consistently corresponded with decreases in the HO-CDI rate. These findings should encourage ACHs to invest efforts into monitoring antibiotic use and targeting unnecessary and inappropriate use across all classes of antibiotics.

Our study, utilizing a new cohort of hospitals during more recent years, continues to identify associations between hospital-level antibiotic use and the HO-CDI rate and suggests that reducing antibiotic use, in conjunction with other infection control and prevention measures, can reduce facility-level rates of HO-CDI.

## Supplementary material

For supplementary material accompanying this paper visit http://doi.org/10.1017/ice.2021.151. click here to view supplementary material
