# Supplementary figures and images for: Associations of facility-level antibiotic use and hospital-onset Clostridioides difficile infection in US acute-care hospitals, 2012–2018
Source: Infect Control Hosp Epidemiol. 2021 May 7;43(8):1067–9. doi: 10.1017/ice.2021.151 (PMC9346446; doi:10.1017/ice.2021.151)

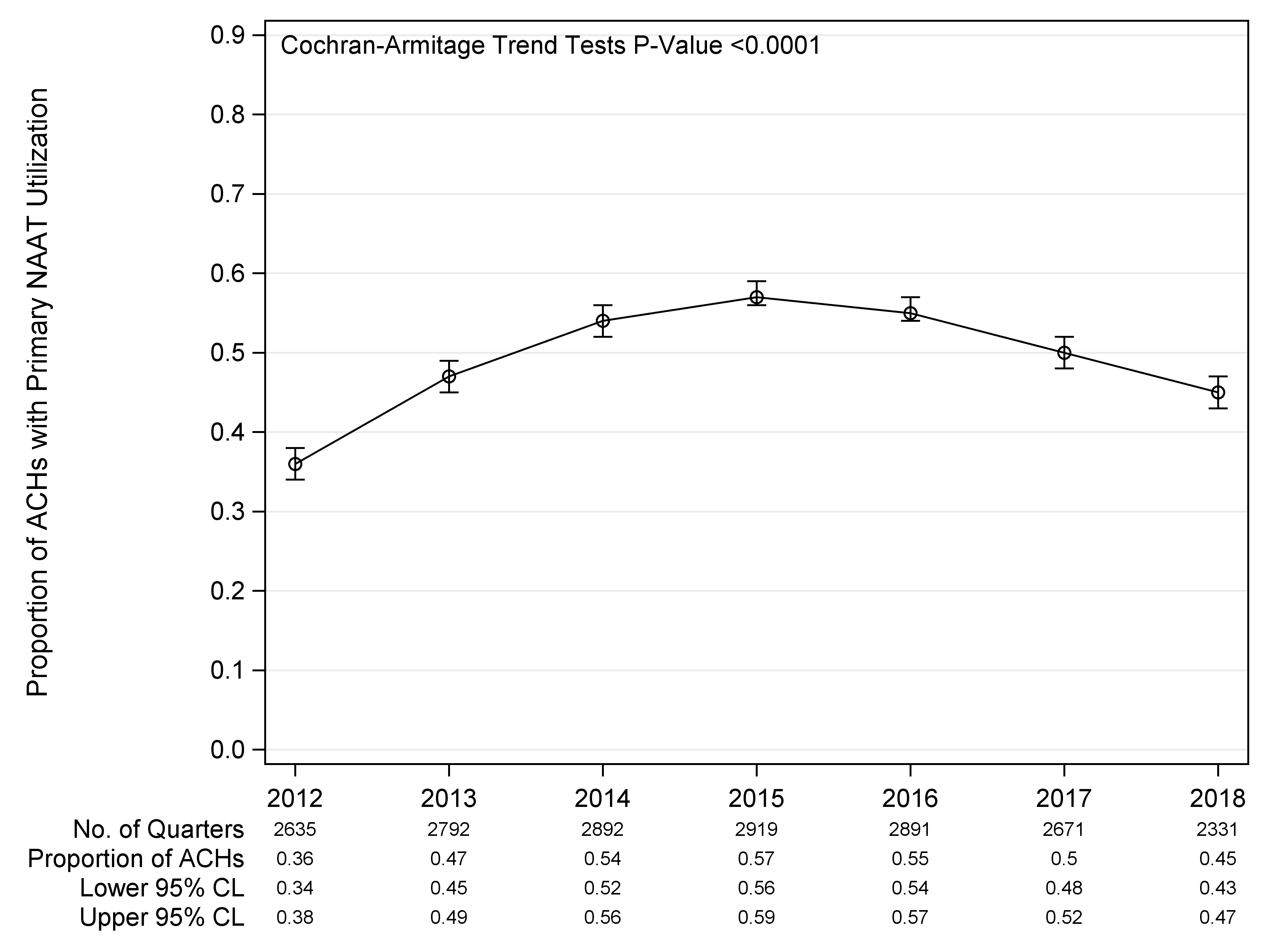

Supplement: Supplementary file 1 [file S0899823X21001513sup.zip › S0899823X21001513sup001.tif]

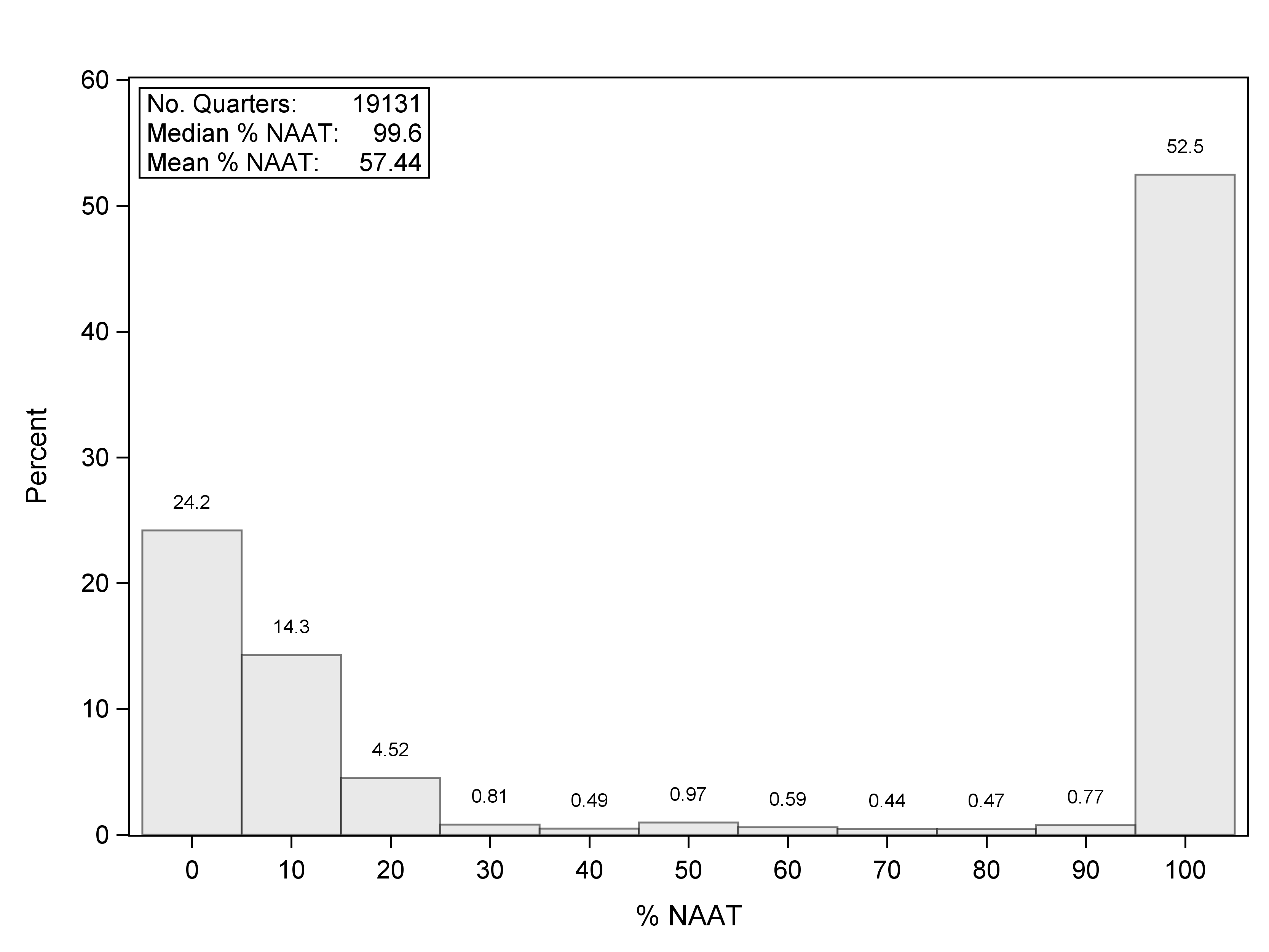

Supplement: Supplementary file 1 [file S0899823X21001513sup.zip › S0899823X21001513sup002.tif]
